# The Cessation in Pregnancy Incentives Trial (CPIT): study protocol for a randomized controlled trial

**DOI:** 10.1186/1745-6215-13-113

**Published:** 2012-07-20

**Authors:** David M Tappin, Linda Bauld, Carol Tannahill, Linda de Caestecker, Andrew Radley, Alex McConnachie, Kathleen Boyd, Andrew Briggs, Liz Grant, Alan Cameron, Susan MacAskill, Lesley Sinclair, Brenda Friel, Tim Coleman

**Affiliations:** 1 Paediatric Epidemiology and Community Health Unit, Section of Child Health, Division of Developmental Medicine, Glasgow University, Yorkhill Campus, Glasgow G3 8SJ, Scotland, U.K; 2 Centre for Tobacco Control Studies, and School of Management, University of Stirling, Stirling, FK9 4LA, Scotland, U.K; 3 Glasgow Centre for Population Health, 1st Floor, House 6, 94 Elmbank Street, Glasgow, G2 4DL, Scotland, U.K; 4 NHS Greater Glasgow& Clyde, J B Russell House, Gartnavel Royal Hospital, 1055 Great Western Road, Glasgow, G12 OXH, Scotland, U.K; 5 Directorate of Public Health, NHS Tayside, Kings Cross Hospital, Clepington Road, Dundee, DD3 8EA, Scotland, U.K; 6 Robertson Centre for Biostatistics. Boyd Orr Building, University Avenue, Glasgow, G12 8QQ, Scotland, U.K; 7 Institute of Health and Wellbeing, University of Glasgow, 1 Lilybank Gardens, Glasgow, G12 8RZ, Scotland, U.K; 8 NHS Greater Glasgow and Clyde, West House, Gartnavel Royal Hospital, Glasgow, G12 0XH, Scotland, U.K; 9 R205 Level 2, Queen Mother’s Hospital Tower Block, Glasgow, G3 8SJ, Scotland, U.K; 10 U.K. Centre for Tobacco Control Studies and NIHR School for Primary Care Research Division of Primary Care, University of Nottingham, Nottingham, NG7 2UH, Scotland, U.K

**Keywords:** Intervention, Maternal and child health, Outcomes, Pregnancy, Prevention, Smoking

## Abstract

**Background:**

Seventy percent of women in Scotland have at least one baby, making pregnancy an opportunity to help most young women quit smoking before their own health is irreparably compromised. By quitting during pregnancy their infants will be protected from miscarriage and still birth as well as low birth weight, asthma, attention deficit disorder and adult cardiovascular disease. In the UK, the NICE guidelines: ‘How to stop smoking in pregnancy and following childbirth’ (June 2010) highlighted that little evidence exists in the literature to confirm the efficacy of financial incentives to help pregnant smokers to quit. Its first research recommendation was to determine: Within a UK context, are incentives an acceptable, effective and cost-effective way to help pregnant women who smoke to quit?

**Design and methods:**

This study is a phase II exploratory individually randomized controlled trial comparing standard care for pregnant smokers with standard care plus the additional offer of financial voucher incentives to engage with specialist cessation services and/or to quit smoking during pregnancy.

Participants (n = 600) will be pregnant smokers identified at maternity booking who, when contacted by specialist cessation services, agree to having their details passed to the NHS Smokefree Pregnancy Study Helpline to discuss the trial. The NHS Smokefree Pregnancy Study Helpline will be responsible for telephone consent and follow-up in late pregnancy. The primary outcome will be self reported smoking in late pregnancy verified by cotinine measurement. An economic evaluation will refine cost data collection and assess potential cost-effectiveness while qualitative research interviews with clients and health professionals will assess the level of acceptance of this form of incentive payment. The research questions are: What is the likely therapeutic efficacy? Are incentives potentially cost-effective? Is individual randomization an efficient trial design without introducing outcome bias? Can incentives be introduced in a way that is feasible and acceptable?

**Discussion:**

This phase II trial will establish a workable design to reduce the risks associated with a future definitive phase III multicenter randomized controlled trial and establish a framework to assess the costs and benefits of financial incentives to help pregnant smokers to quit.

**Trial registration:**

Current Controlled Trials ISRCTN87508788

## Background

Smoking during pregnancy increases miscarriage and stillbirth, accounting for up to 4000 UK deaths annually, and pre-term birth and low birth weight leading to perinatal morbidity [1]. A third of excess stillbirths and post-natal deaths associated with living in deprived areas are explained by smoking during pregnancy [2]. In Scotland 70% of women have a baby [3], making pregnancy an opportunity to help most women quit before their health is permanently compromised. In 2009, 24% of women booking for maternity care self-reported as current smokers [4]. Less than 1 in 20 of these women quit [5]. A study in the USA [6] in 2001 calculated the additional first year costs to health services as $1200 per smoker. Smoking is a major preventable cause of premature mortality for the mother, and lifelong benefits of quitting for children include reduced incidence of asthma and attention deficit disorder.

Following the 1998 White paper ‘Smoking Kills’, evidence-based National Health Service (NHS) smoking cessation services using behavioral support were developed [7-9] and now exist throughout Scotland [5]. Most of these services aim to provide tailored support (referred to in this paper as NHS Smokefree Pregnancy Services - NSPS) to pregnant women on a one-to-one basis, and evidence suggests that such interventions during pregnancy are effective [10]. Nicotine replacement therapy (NRT) is also offered, although its efficacy in pregnancy is not yet clear [10]. In Greater Glasgow & Clyde (GG&C), with 15,000 pregnancies per year, pregnant smokers identified at maternity booking are currently routinely referred to the NSPS on an ‘opt-out’ basis, without consent for transfer of contact information [7]. This practice is in keeping with the recent National Institute for health and Clinical Excellence (NICE) guidelines in the UK [11]. Women are then contacted by NSPS advisers who ask if they would like to quit and offer a face-to-face appointment in a clinic or home setting. If the client attends they set a quit date and a risk assessment for use of NRT during pregnancy is performed.

Participation figures for areas with recognized specialist smoking cessation in pregnancy services from a Scotland-wide audit are shown below [5]:

A. Estimated number of pregnant smokers at maternity booking [12], 8170 (100%).

B. Smokers identified by self report at routine booking questionnaire, 6128 (75%).

C. Smokers referred to specialist pregnancy cessation services, 3188 (39%).

D. Smokers engaged - face to face contact with specialist cessation service, 763 (10%)^a^.

E. Smokers who ‘set a quit date’, 779 (10%).

F. Smokers who had quit 4 weeks later, 236 (3%).

^a^Different denominator.

In Scotland, all women who book for maternity care are asked if they are current, former or never smokers (B above). At present 25% deny their habit (A to B) [12]. This problem is being addressed by routinely testing all pregnant women at maternity booking for carbon monoxide using a breath test [11]. Only half were referred (B to C) due to busy clinics and perceived client disapproval. Only 10% reached specialist cessation services such as those available in Glasgow (D). Nearly all of these ‘set a quit date’ (E) and 3 in 10 (F) quit. Some improvement (E to F) may be possible as 4 in 10 quit [13] with support from NHS cessation services in other parts of the UK.

Incentives are thought to increase participation and engagement [14,15], retention [16,17], and cessation [15-18]. If offered incentives, women who deny smoking may report as current smokers (A to B). Clients may self-refer or ask to be referred (B to C). With incentives, a greater proportion may engage (C to D) [5]. Improvement in the numbers quitting (D to F) will follow. In Scotland, when more pregnant smokers were referred, more engaged and more quit [5].

**Figure 1:**
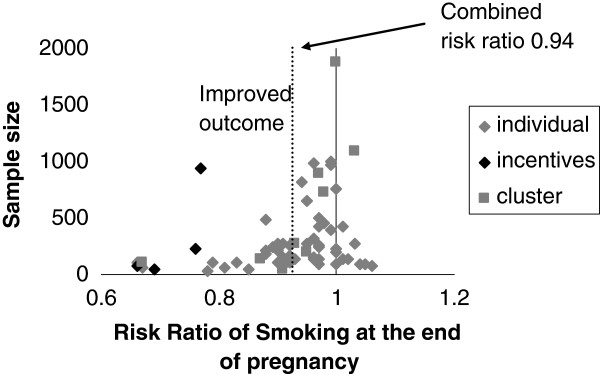
**Funnel plot.** Figure 1 is a funnel plot of all studies included in the Cochrane systematic review: interventions for promoting smoking cessation during pregnancy [10]. The overall risk ratio for smoking cessation in each study is plotted on the x axis. The sample size of each study is plotted on the y axis. The plot illustrates possible publication bias with a gap caused by ‘missing’ studies upsetting the symmetry on the right hand side of the figure, indicating that small negative studies may not have reached the point of publication. Diamond shaped symbols indicate individual randomization whereas square symbols indicate that cluster randomization was used in the trial. The four black diamond shapes on the left side represent the four incentives trials included in the review. The dashed vertical line crosses the x axis at the weighted mean risk ratio of smoking reduction in all studies comparing intervention with control – 0.94 (95 % confidence interval 0.93 to 0.95).

A 2009 Cochrane systematic review of interventions for promoting smoking cessation during pregnancy [10] asked: Do interventions improve quit rates? Are some interventions better than others? Figure 1 is a funnel plot describing the outcome from all included trials showing intervention better than control to the left. Fewer small trials on the right (control better than intervention) indicates some publication bias so the pooled risk ratio 0.94 may overestimate any combined positive effect. The four incentives trials showed significantly better outcomes than other interventions: pooled risk ratio 0.76 (95% confidence interval (CI) 0.71 to 0.91).

However, the incentives trials are all from the USA. The first trial was conducted 25 years ago and only included incentives as a minor part of an extensive intervention. Other incentives trials were small [16-18] and did not have a ‘no incentive’ control group [17,18]. None attempted to measure change in participation or engagement with an intervention. The NICE guidelines [11] issued in June 2010 highlighted that little evidence exists in the literature to confirm the efficacy of financial incentives to help pregnant smokers to quit. Its first research recommendation was: “Within a UK context, are incentives an acceptable, effective and cost-effective way to help pregnant women who smoke to quit?”

Since 2007, NHS Tayside in Scotland has piloted an incentives model - £12.50 per week of supermarket vouchers - to reward smoking cessation from quit date to 3 months after birth. The total payment for quitters is up to £650. The project has no formal contemporaneous control group but the number of women accessing cessation support has increased since incentives were introduced [19]. Women access the service at their local pharmacies, which provide NRT as well as support and validate whether the client has stopped smoking. This information initiates a payment onto a store card. During 2009, 169 (14%) of about 1200 pregnant smokers [5] joined the program (the equivalent of engagement) and 94 (8%) women had quit smoking 4 weeks later; 42 were still quit at delivery (personal communication, Andrew Radley). This cessation rate is higher than those obtained by other Scottish smoking cessation in pregnancy services, where the average quit rate at 4 weeks is 3%, and the best 5% [5]. A feasibility study for an incentives trial in Glasgow reported in January 2009 [20]. It explored how the Tayside program could be adapted for service configuration in Glasgow. The feasibility study provided the foundation for development of this trial.

This phase II trial will provide the tools required to run a future appropriately powered definitive phase III multicenter randomized controlled trial. The aim of that future phase III trial will be to establish whether or not incentives increase engagement with services, quit attempts and successful cessation to provide a more cost-effective pregnancy smoking cessation service model generalizable to the whole UK and beyond.

## Design and methodology

### Population

In 2010 there were 58,051 births in Scotland with 14,155 (24%) to mothers resident in NHS GG&C Health Board area. Twenty-six percent of births in Scotland are to women who live in the most deprived quintile, whereas in GG&C it is 44% [21]. Of the most deprived women who were asked, 35% self reported that they smoked at maternity booking compared with 7.1% of the least deprived [22]. Overall, in GG&C 25% of women self report as current smokers at maternity booking.

### Design

**Figure 2:**
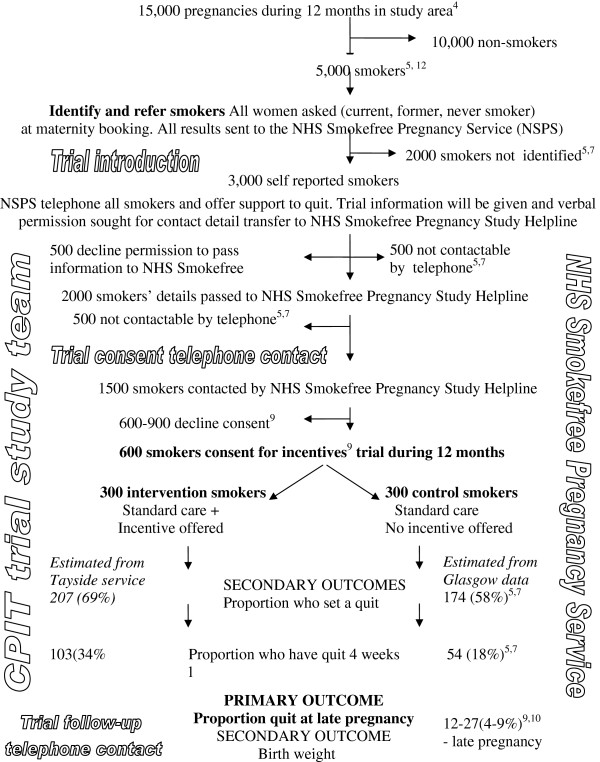
**Flow diagram for Cessation in Pregnancy Incentives Trial (CPIT).** Figure 2 projects the flow of pregnant women through the trial over a 12-month period. In the NHS Greater Glasgow & Clyde study area, 15,000 pregnancies are recorded at maternity booking; 5,000 are to smokers, some who conceal their habit, and 3,000 are identified at maternity booking by self-report as current smokers. Their information is routinely passed to NHS Smokefree Pregnancy Services (NSPS) using an automated system. Attempt is made to contact all self reported smokers. We estimate that 500 will not be contactable by NSPS and that 500 will decline permission for their details to be passed to the NHS Smokefree Pregnancy Study Helpline (Study Helpline). Once details have been passed, 500 will not be contactable leaving 1,500 contacted by the Study Helpline. We expect that 600 to 900 will decline consent leaving 600 to 900 who agree to enroll in the study over 12 months. Of these, 300 will be randomly allocated to the intervention group to be offered incentive payments as well as standard care to stop smoking during pregnancy and 300 will be randomly allocated to receive the offer of standard care without incentive payments to quit smoking.

The study is designed as a phase II single blinded (outcome assessor blind to randomization status at follow-up telephone contact) individually randomized controlled trial. The procedures are illustrated schematically in Figure 2 and detailed in the text.

### Research questions

#### *Trial*

1. What is the likely therapeutic efficacy of financial incentives to encourage women to attend specialist cessation services, to set a quit date, to quit smoking and be abstinent at 34 to 38 weeks gestation towards the end of pregnancy?

2. Is individual randomization an efficient trial design without compromising validity through bias due to differential follow-up of disaffected control arm participants?

#### *Health economic study*

3. Are incentives potentially a cost-effective means of helping pregnant women to stop smoking?

#### *Qualitative study*

4. Can incentives be introduced in a way that is feasible and acceptable to women and service providers and are there any unintended consequences?

### NHS Smokefree Pregnancy Services Greater Glasgow & Clyde Health Board area

At their maternity booking appointment pregnant women in NHS GG&C are asked for consent for screening which includes procedures to identify and offer treatment to current smokers – self report and routine carbon monoxide breath test with follow-up by NSPS. All women with either a carbon monoxide level of 5 ppm and above or self reported as current smokers (at least 1 cigarette in the last week) are notified to NSPS using a rapid electronic system. NSPS advisers contact these women by telephone and offer a smoking cessation counseling service which utilizes ‘Withdrawal Oriented Therapy’ [23]. Support includes a face-to-face appointment, weekly telephone support for 4 weeks and the offer of NRT dispensed by local pharmacies free of charge to support a quit attempt for up to 12 weeks.

### Trial inclusion criteria

Participants must satisfy the following criteria to be included in the study:

· age at least 16 years and pregnant less than 24 weeks gestation at maternity booking.

· self report as current smokers.

· have a carbon monoxide breath test result of 7 ppm or greater - NICE guideline level for referral of pregnant smokers – PH26 [11].

· live in the NHS GG&C Health Board area.

· give permission for their contact details to be passed to NHS Smokefree Pregnancy Study Helpline.

· be able to understand and speak English in order to provide verbal telephone consent to take part in the trial.

### Screening

Early in the first telephone contact call from an NSPS adviser, eligible smokers will be given information about the trial and asked to give their verbal permission for contact details to be passed to the NHS Smokefree Pregnancy Study Helpline so that formal trial telephone consent procedures can take place. The Caldicott Guardian has approved this verbal permission required to pass this contact information. Those who give permission for contact details to be passed will be sent a letter and a trial information sheet outlining the nature and importance of the trial. The NSPS adviser will then continue with standard treatment and follow-up procedures.

### Recruitment

We will enroll 600 eligible pregnant smokers to this phase II trial over a 12 month period. This is illustrated in Figure 2 based on existing throughput from local services in the study area and is a conservative time period taking into account smokers not identified or referred and those who decline consent to take part in the incentives trial. We expect high enrollment of eligible smokers and that knowledge of the trial will increase eligible smokers who agree to consider cessation during this pregnancy and consent to take part in the trial.

### Consent and randomization procedure

At least 7 days after the first NSPS contact call, eligible smokers who allow their contact details to be passed on will be contacted by the Helpline to undergo formal consent procedures. Telephone contact will be attempted on three occasions at the time slot preferred by the client - weekday, evening or weekend - after which no further attempts at enrollment will be made. During the consent call potential participants will be fully informed about the nature and relevance of the trial, and exactly what will be involved if they agree to take part. Early in the consent call potential participants will be asked to provide information not asked for at maternity booking including questions measuring level of addiction to nicotine and partner smoking habits. Audio recordings of the consent process will be stored in accordance with Good Clinical Practice guidelines. Eligible smokers who consent to take part in the trial will be sent a written copy of their consent form. Individuals who give informed consent will be enrolled in the trial and randomized. The random allocation will be fed back to the participant. The Helpline adviser cannot influence or predict the random allocation which is integrated into the clinical information system. It will also provide a concealed (not available to the NHS Smokefree Pregnancy Study Helpline adviser during the consent call) random date between 34 and 38 weeks gestation for this pregnancy when the Helpline will contact the participant again to ascertain the primary outcome measure of self reported smoking during the last 8 weeks which will be corroborated by cotinine measurement.

### Baseline (pre-randomization) data collection

#### *Identification and contact*

Patient name, address, full postcode, telephone numbers (home, mobile, office).

Best time to call, best number, estimated date of delivery, name of referrer, General Practitioner name.

#### *Eligibility criteria*

Self reported smoking status at maternity booking, carbon monoxide breath test level at maternity booking, date of carbon monoxide reading, age at first telephone contact, English speaking, gestation at booking, consent, copy of consent form sent.

#### *Smoking addiction level and other*

Fagerstrom questions [24] score out of 10, parity, partner smokes.

### Randomization

Randomization will be embedded within the trial database. The random allocation sequence will be generated by the Robertson Centre for Biostatistics, University of Glasgow (part of the Glasgow Clinical Trials Unit), using the method of randomized permuted blocks, with a block length of 4. In addition, a random date between 34 and 38 weeks gestation for each pregnancy will be generated as the date for primary outcome data collection. Patient details will be entered prior to randomization so that the allocation is concealed from Helpline staff and the client until after the patient has been enrolled in the study and provided baseline information. The participant will be informed of their random allocation group towards the end of the Helpline ‘trial consent’ telephone contact call, but will not be informed of the date when their primary outcome data will be collected.

For a period during trial recruitment an extra consent question will be ‘turned on’ during the consent call asking clients also to consent to being contacted by trial staff for the purposes of one-to-one interviews to fulfill qualitative aspects of this research trial.

#### *Voucher incentive payments*

Those allocated to the intervention group will be offered financial incentives - Love2Shop vouchers - that can be redeemed in a wide array of UK shops but not used for the purchase of cigarettes or alcohol. The total amount available is £400 if women remain abstinent at each monitoring point. Women will receive incentive payments outlined below:

£50 for attending a face-to-face appointment with their NSPS adviser and setting a quit date;

£50 if quit 4 weeks after their quit date corroborated by a carbon monoxide breath test result less than 10 ppm collected by a research nurse;

£100 if quit after 12 weeks corroborated by a carbon monoxide breath test collected by a research nurse;

£200 if they self report quit for at least 2 months when contacted for primary outcome assessment by the Helpline at 34 to 38 weeks gestation. This will be corroborated by a carbon monoxide breath test result less than 10 ppm collected by a research nurse.

### Trial participation payments

In order to minimize loss to follow-up, particularly among control participants, £25 will be given to all participants if they provide primary outcome information (and required samples to corroborate abstinence) at 34 and 38 weeks gestation.

### Quantitative data collection

#### *Primary outcome*

*Smoking status at 34 to 38 weeks gestation, towards the end of pregnancy*. Follow-up telephone contact will be attempted by NHS Smokefree Pregnancy Study Helpline at a random date between 34 and 38 weeks gestation allocated at the time of the initial randomization. A patient record check will be made by the NHS Smokefree Pregnancy Service administrator looking for stillbirth, miscarriage or patient death the week prior to the telephone contact at 34 to 38 weeks gestation. Miscarriage or stillbirth may not preclude a woman from receiving vouchers if they are in the intervention group.

Three attempts will be made to contact participants. If no contact is possible the participants will be followed up by the research nurse either by telephone or by a home visit after a contact detail check with the participant’s General Practitioner. If participants self report as non-smokers then a research nurse will make an appointment to collect urine and saliva for cotinine estimation (as well as a breath test for carbon monoxide to trigger the incentive payment).

If successful contact is made, the participant will be asked: ‘Have you smoked in the last 8 weeks?’ If yes ‘Have you smoked more than 5 cigarettes in that time?’ Self report will be corroborated by cotinine estimation on saliva and urine. Urine (cut-off 44.7 ng/ml) or saliva (cut-off 14.2 ng/ml) cotinine will be measured by ABS laboratories limited (abslabs@biopark.org.uk).

The important aspect of the primary outcome for this phase II trial is the proportion of participants successfully followed up from the intervention but particularly the control group. If acceptable levels (about 90%) of participants can be successfully followed up with a similar proportion in the intervention and control groups, then individual randomization can be accepted as a design choice for a future phase III trial that does not produce significant bias due to differential loss to follow-up between groups.

#### *Secondary outcomes*

Engagement with specialist pregnancy cessation services is achieved if the participant arrives at a first face-to-face appointment with the NSPS adviser and sets a quit date.

Quit at 4 weeks after quit date will be ascertained with the question ‘Have you smoked in the last 2 weeks, even a puff?’ at routine telephone contact by the NSPS adviser. This will be corroborated by a carbon monoxide breath test collected at routine pharmacy visits to collect NRT. These data will be available from both intervention and control groups.

##### *Birth weight*

The best methods to gather these data efficiently will be examined and tested. Maternal height and weight is recorded routinely at maternity booking and will be used to help clarify birth weight differences during data analysis. Babies of smokers are on average 300 g (10%) lighter than babies born to non-smokers [25].

##### *Unintended consequences*

We do not anticipate the provision of extra finance to participants will have any adverse effects. We are not systematically documenting adverse events that happen during pregnancy. We are, however, examining unintended consequences formally using in-depth interviews with a sample of enrolled clients. Such possible unintended consequences include, for instance, taking up smoking prior to maternity booking in order to receive incentive payments by stopping afterwards.

##### *Health economics*

Economic analysis will be undertaken alongside the trial, utilizing the costs, resource use and effectiveness data generated within the trial. For example, the cost of financial incentives, NRT, service delivery, and so on, will be collected and combined with resource use data. The number of quitters at the end of the trial will be combined with the cost data to calculate the incremental cost per late pregnancy quitter. The secondary outcome measure of child birth weight is strongly related to health care costs. Birth weight will be used to determine any difference in weight and therefore health care costs between neonates of mothers in the intervention group compared to mothers allocated to the control group.

##### *Health economic assessment*

A within-trial analysis will explore the incremental cost per late pregnancy quitter, followed by a longer term analysis incorporating the cost to the NHS of any difference in birth weight due to smoking and looking at potential lifetime quality-adjusted life year (QALY) gains to the mother and child.

A health economic model we previously developed [26] (which uses a Markov design to model the lifetime likelihood and impact of cessation, expressing the long-term health benefits of quitting smoking in terms of QALYs saved and the potential long-term reduced costs to the health service of cessation) will be used. This model will be adapted to incorporate the trial information and relevant published evidence in order to capture the short and longer term costs and health gains for both mother and child of smoking cessation during pregnancy. The outcomes will be presented in terms of the incremental cost per QALY saved. The model will be analyzed probabilistically in order to characterize uncertainty in the parameters, and estimate confidence limits around the cost and effectiveness outcomes. Areas of uncertainty identified within the model will help inform decisions about the potential value of future research. This model will ascertain whether any (and what type of) data should be collected to inform a more definitive economic evaluation and contribute to the optimal design of a Phase III trial.

### Analysis

Analysis of outcomes will be by intention-to-treat, as the intervention is the offer of financial incentives to engage with cessation services and to quit smoking.

Differences by subgroup (maternity unit, deprivation score, age group, and so on) will be explored.

### Sample size

The study is not designed to be able to detect a specified difference in the primary outcome of quit rate at 34 to 38 weeks gestation. The main objective is to inform the feasibility and design of a future definitive trial. However, assuming a quit rate of 4% in the usual care arm of the study, then with 600 participants the study will have 90% power to detect an increase in quit rates to 11.4%, or 80% power to detect an increase to 10.2%, based on a continuity-corrected *χ*^2^ test at a 5% level of significance. Even if these outcome rates were achieved in this study, a Phase III study would be required to demonstrate that the intervention can be effective in other settings.

### Overview of qualitative research components

#### *Qualitative outcomes*

The qualitative research will take the form of a process evaluation designed to inform aspects of the proposed trial. It will contribute to:

· assessing the feasibility and acceptability to women and service providers of using incentives to promote cessation during pregnancy (including identification of barriers and facilitators).

· identifying any unintended consequences of participation.

· exploring responses to randomization approaches

· providing guidance on how the intervention might be modified for the Phase III randomized controlled trial.

Interviews will be conducted with small samples of (i) pregnant women and (ii) relevant professionals by researchers at Stirling University led by Susan MacAskill. This work will expand on qualitative research already undertaken with clients and professionals in relation to ‘Give it up for Baby’, the incentives scheme in Tayside [19].

Qualitative interviews will be conducted with pregnant women and professionals using semi-structured topic guides developed in line with the research aims. Topics will not be explored in a prescriptive manner but as part of an open discussion. This flexible format will enable additional salient topics and insights to emerge. In broad terms, the focus for the different respondent groups will be as follows:

One-to-one interviews with pregnant women (all of whom have engaged with the service) will explore views on issues around the delivery and promotion of the cessation service, response to incentive features and randomization, and any unintended consequences. Those interviewed prior to the trial start will be asked to respond to the concept of the incentives and the trial elements in addition to routine service elements, whilst those interviewed during the trial will also be asked to respond to these elements but based on personal experience of the trial.

Focus group interviews with pregnant women (smokers/recent ex-smokers) interviewed before the trial starts will also explore views on issues around the delivery and promotion of the cessation service, response to incentive features and randomization, and any unintended consequences. Responses can incorporate personal experiences, but will largely be based on response to the concept of these issues.

Interviews with professionals will explore issues around implementation of the intervention and the trial elements, identify challenges and ways they have been overcome, and perceived response among participants.

With the participants’ consent, all interviews and focus group discussions will be recorded as digital audio files, which will then be transcribed in full for thematic analysis. Transcripts will be organized using a thematic framework based on topics specified in the topic guide and emerging themes identified through a process of familiarization with transcript texts.

#### *Sample and recruitment for the qualitative element*

Prior to the trial starting, up to 10 pregnant smokers will be recruited from amongst those already in contact with NSPS in Glasgow. Women will be informed about the study by specialist advisers on smoking in pregnancy and asked if they are interested in participating. Those interested will be provided with an information sheet and will be asked if their details can be passed to the lead qualitative researcher. Those who consent to their details being sent on will be contacted by phone to address any queries and to arrange an interview time and place. Alternatively, if they consent at the time of the initial information being provided, interviews can be conducted following their second session with the specialist advisor. Interviews will be conducted face to face and written consent obtained.

In addition, two one-off focus groups (4 to 8 respondents each) with pregnant women (incorporating smokers/recent ex-smokers) will be conducted. Women will be recruited by the researcher through ante-natal classes or other appropriate groups attended by pregnant women and again explanations will be given and consent sought to participate.

During the trial an additional 20 women will be interviewed after they have engaged with the trial. These will be different women from those interviewed prior to the trial. Pregnant smokers will be informed about this part of the study by the NHS Smokefree Pregnancy Study Helpline and asked if they are interested in participating in the interviews as part of the initial consent process. Details of those who express an interest and consent to be contacted will be passed to the research team. Points at which individuals will be interviewed will be spread across the pregnancy (that is, at around 4, 12 and 20 weeks post-set quit date). Mechanisms will be in place to ensure there are no contraindications prior to re-contacting those to be interviewed in later pregnancy (for example, any adverse pregnancy outcomes such as miscarriage). Both continued participants and those who have dropped-out will be included in the sample.

Respondents will be recruited from two hub areas in Glasgow and Clyde. All pregnant women participants will be offered £20 in recognition of their contribution to the study.

Professionals will also be interviewed as part of the qualitative study. They will be approached through appropriate organizational structures, facilitated by study co-applicants. Information sheets will be provided and consent obtained. The professional sample is intended to reflect a range of perspectives on implementation and the conduct of the trial. They will therefore include: pregnancy smoking cessation advisor(s) and manager, Clinical Research Facility nurse(s), interviews with the NHS Stop Smoking Study Helpline staff/managers, community midwives and manager, and community pharmacy facilitator(s). Professionals will be interviewed by telephone or face to face.

## Discussion

In Scotland, up to a third of pregnant women smoke throughout pregnancy. This is responsible for a third of miscarriages, still births and sudden infant deaths during the first year of life. There would be significant long-term health gains for mother and child if smokers quit during pregnancy. At present, less than 10% of pregnant smokers take up the offer of free smoking cessation services and only 3% quit during pregnancy. Modest incentive payments to engage with cessation services and/or to quit smoking may provide a substantial benefit by decreasing pregnancy health care costs and first-year health care costs. If women stay smoke-free, long-term health care costs will be substantially reduced. The results of this phase II trial will reduce the risks associated with funding a definitive phase III multicenter trial. The phase III trial will examine the costs and benefits of providing financial incentive payments for smoking cessation during pregnancy.

## Ethics approval

Ethics approval was received from West of Scotland REC2 on 25 May 2011.

Caldicott guardian approval was given on 3 February 2011 to allow NSPS GG&C to pass information to the NHS Smokefree Pregnancy Study Helpline if the client gives verbal permission that contact details can be passed.

A major amendment to the trial protocol was approved on 6 March 2012 to allow use of anonymized qualitative interview data by the Health Technology Assessment Programme 10/31/02 BIBS: Benefits of Incentives for Breastfeeding and Smoking cessation: A platform study for a trial.

## Trial organization

### Trial Steering Committee

The overall scientific aspects of the project will be managed by a Steering Committee. The Steering Committee will include the investigators above. The Steering Committee will take all executive decisions.

The responsibility of the Steering Committee is to ensure the scientific integrity and quality of the project. To achieve this, the specific responsibilities of the Steering Committee include: maintaining adherence to the study protocol; approving changes to study protocol if required; reviewing quality assurance indicators; monitoring study recruitment and the overall study timetable; advising, as required, on specific scientific items that may arise; compliance with legislation; adherence to research governance; reporting to funders; approving publication and dissemination strategies.

The Steering Committee will meet every 6 months.

### Project Management Group

A Project Management Group (comprising as required principal investigators, a trial manager, a trial administrator, a senior manager from NSPS, a data manager from NHS Research and Development, a statistician, a senior representative from NHS Stop Smoking Study Helpline, a senior manager from NHS GG&C Research and Development, a health economist, a senior qualitative researcher) will run the trial on a day-to-day basis to ensure the smooth operation of the project. Review meetings will be held with other members of the team as appropriate.

The responsibilities of the Project Management Group include: establishing and monitoring recruitment of participants; distributing and supplying data collection forms and other appropriate documentation for the trial; data collection and management; data entry and cleaning; data analysis; organizing and servicing the Trial Steering Committee.

### Data Monitoring Committee

An independent Data Monitoring Committee will not be established as adverse events related to the financial incentives intervention are not envisaged and are not being systematically collected.

### Data Co-ordinating Centre

The Data Co-ordinating Centre will be based at the Paediatric Epidemiology and Community Health Unit, Glasgow University. The responsibilities of the Data Co-ordinating Centre are to set up and run systems for data entry, data verification and the checking of errors and overdue data reports.

### Callcredit plc

Callcredit plc, Leeds UK, will design and build the web based portal for data entry comprising the main Electronic Data Capture system. This system will allow NHS and research staff to enter data in a timely fashion triggering voucher incentive payments dispatched by Callcredit.

### NHS Stop Smoking Study Helpline

NHS Stop Smoking Study Helpline will provide call centre services: the consent call for participant enrollment, and the final call to determine the primary outcome (self reported smoking at 34 to 38 weeks gestation).

### NHS Research and Development Glasgow & Clyde

NHS Research and Development are supporting this trial by providing extra research nursing support for participant enrollment and follow-up and project assistance for data entry.

### The Robertson Centre for Biostatistics, University of Glasgow

The Robertson Centre is part of the Glasgow Clinical Trials Unit, and will provide statistical analysis and reporting for the study.

### NHS Smoking in Pregnancy Services

NSPS GG&C are discussing the trial with participants and asking permission for contact data to be passed outwith the NHS to The Listening Company Glasgow Scotland (a call centre with extensive experience working with the NHS and who are known for the trial as the NHS Stop Smoking Study Helpline) for the consent call. NSPS staff are providing an encrypted list of potential participants to the NHS Stop Smoking Study Helpline on a weekly basis. They are providing cost data for the economic analysis.

### Publication policy

The primary results of the trial will be published with authorship in relation to specific participation in the study, with the name order to be presented by the principal investigators for consideration by the Trial Steering Committee. Suggested revisions in order of authors should meet with the approval of the principal investigators. Publications in specific areas of the study or on methodological aspects can be led by co-investigators in their area of expertise subject to approval by the Trial Steering Committee and the principal investigators. The requirements for authorship will follow recommended practice in journal guidelines.

### Confidentiality

Encryption defined by NHS GG&C security management will be in place to pass data for potential participants from NSPS to NHS Smokefree Pregnancy Study Helpline, a process agreed by the Caldicott Guardian. Both NHS Smokefree Pregnancy Study Helpline and CallCredit plc have long histories of managing Government-related services and are able to demonstrate their commitment to data security and quality management through their ISO27001 and ISO9001 accreditations. Their ISO27001 accredited Information Security Management Systems demand that all of their systems and processes are maintained with confidentiality, integrity and availability of data at the core. In addition both companies are ISO9001 accredited, the internationally recognized standard for Quality Management Systems. During and after data analysis participants will be identified by their trial number to ensure confidentiality.

## Trial status

Recruitment started in December 2011. On 9 june 2012, 199 of 600 were enrolled in the trial.

## Abbreviations

CI: confidence interval; CPIT: Cessation in Pregnancy Incentives Trial; GG&C: Greater Glasgow & Clyde; NHS: National Health Service; NICE: National Institute for health and Clinical Excellence; NRT: nicotine replacement therapy; NSPS: NHS Smokefree Pregnancy Services; QALY: quality-adjusted life year.

## Competing interests

The authors declare that they have no competing interests.

## Authors’ contributions

DT and LB conceived the study. DT, LB, CT, LDC, AB, AC, LG AR, SM, AM and TC were applicants for the funding. All authors were involved in designing the study and drafting the protocol. KB and AB designed the health economic aspects of the study. SM designed the qualitative aspects of the study. All authors read and approved the final protocol.

## Authors’ information

1. *David Tappin* (Co-Principal Investigator): Professor of Clinical Trials for Children based at the Paediatric Epidemiology and Community Health Unit. Will co-ordinate and manage the overall running of the trial and will be closely involved in data analysis and paper writing and dissemination of results.

2. *Linda Bauld* (Co-Principal Investigator): Professor of Socio-Management Stirling Management School and U.K. Centre for Tobacco Control Studies. Has extensive experience of quantitative and particularly qualitative work related to smoking cessation. She will support the trial on a day to day basis and be involved in paper writing and dissemination of results

3. *Carol Tannahill*: Director of the Glasgow Centre for Population Health. She will support the trial and give guidance.

4. *Linda de Caestecker*: Director of Public Health Greater Glasgow and Clyde Health Board. She will support the trial set-up and liaison with NHS Smokefree Pregnancy Services and NHS Research and Development, Greater Glasgow and Clyde.

5. *Andrew Radley*: Public Health Pharmacist Tayside. Established ‘Give it up for baby’ an innovative service development providing financial incentives for pregnant smokers to quit in Tayside. He will provide advice from his experience providing financial incentives to help pregnant smokers to quit.

6. *Alex McConnachie*: Assistant Director of Biostatistics, Robertson Centre for Biostatistics has extensive experience of data analysis and study design for clinical trials. He will be responsible for analyzing the final dataset.

7. *Kathleen Boyd:* A health economics research fellow who has planned and will undertake the health economic evaluation.

8. *Andrew Briggs*: Professor of Health Economics has extensive experience investigating the health economic effects related to clinical trials. He will be responsible for the health economic aspects of the trial.

9. *Alan Cameron*: Professor of Obstetrics has been involved in many studies related to obstetric care. He will have responsibility for the obstetric service aspects of the project.

10. *Liz Grant*: Runs the pharmacy aspects of smoking cessation services for NHS Greater Glasgow and Clyde Health Board

11. *Susan MacAskill*: Is a senior qualitative researcher with extensive experience of assessing smoking cessation intervention strategies. She will be responsible for the qualitative aspects of the study.

12. *Lesley Sinclair* (Trial Manager): Has experience of data management and the management of clinical trials. She will run the trial on a day to day basis.

13. *Brenda Friel:* Is health improvement senior, Smokefree Services, who runs the NHS Smokefree Pregnancy Services in Greater Glasgow and Clyde. She has been pivotal in ensuring that this trial runs well. She has been involved in planning and supporting all aspects of the trial process.

14. *Tim Coleman*: Is a senior researcher who has run trials of smoking cessation interventions during and outwith pregnancy. He will provide an overview for the other investigators.
